# Effect of project-based experiential learning on the health service delivery indicators: a quasi-experiment study

**DOI:** 10.1186/s12913-020-4949-5

**Published:** 2020-02-26

**Authors:** T. Chelagat, G. Kokwaro, J. Onyango, J. Rice

**Affiliations:** Institute of Healthcare Management, Strathmore University Business School, Strathmore University, Nairobi, Kenya

**Keywords:** Leadership training, Health service indicators, Team-coaching, Effectiveness, Projects

## Abstract

**Background:**

Kenya’s new constitution passed in 2010 recognizes the right of quality care resulting in the devolution of health service delivery to the sub-national units called counties in 2013. However, the health system performance continues to be poor. The main identified challenge is poor health systems leadership. Evidence shows that addressing health system leadership challenges using different leadership intervention models has the potential to improve health outcomes. The purpose of this study is to report findings on the effect of project-based experiential learning on the health service delivery indicators addressed by 15 health management teams from 13 counties in Kenya, as compared to the non-trained managers.

**Methods:**

A quasi-experimental design without a random sample was used to evaluate the effectiveness of the leadership program**.** The health managers from the 13 Counties and 15 health facilities had previously undergone a 9-month leadership training, complimented with facility-based team coaching based on 15 priority institutional service improvement projects at the Strathmore University Business School. Pre-test and post-test data were collected in three-point periods (beginning, end of the training, and 24-to-60 months post-training). The control group comprised 14 other health institutions within the same counties.

**Results:**

Leadership training and coaching built around priority institutional health service improvement projects in the intervention institutions showed: a) skilled birth attendance increased, on average, by 71%; b) full immunization of children, increased by 52%; c) utilization of in and out-patient services, which on average, increased by 90%; d) out-patient turn-around time reduced on average by 65% and; e) quality and customer satisfaction increased by 38.8% (in all the intervention facilities). These improvements were sustained for 60 months after the leadership training. In contrast, there were minimal improvements in service delivery indicators in the comparison institution over the same period of time. Ninety-three percent of the respondents attributed team-coaching built around priority institutional health service improvement projects as a key enabler to their success.

**Conclusions:**

The study provides support that an intervention underpinned by challenge driven learning and team coaching can improve a range of health service delivery outcome variables.

## Background

Kenya promulgated a new constitution in 2010 [1]. The constitution introduced a devolved system of government that transferred health service management functions from the central government to 47 semi-autonomous government units known as counties in 2013 [2]. The national government is responsible for setting health care standards, the provision of technical support to county governments, and the management of national referral hospitals [3]. Counties became responsible for health service delivery, and the national government’s new focus of responsibility became research and policy. Health service delivery and management functions at the County level are overseen by the County Departments of Health governed by the County Health Management Teams (CHMT). Devolution was meant to foster improved health service delivery with an emphasis on improving, access, utilization and equity [4–6].

Kenya remains one of the countries within sub-Saharan Africa that exhibit insufficient progress in improving its health indicators. The 2014 Kenya Demographic and Health Survey report shows that Kenya had made commendable advances towards child survival in the last 5 years prior to the survey. Findings reported a remarkable decline of childhood deaths to 52 per 1000 live births, compared from the earlier statistics of 74 per 1000 live births as per the 2008–09 KDHS report. There was an improvement in the maternal mortality ratio reported at 362 maternal deaths per 100,000 live births for approximately 7 years prior to the survey [7]. Despite these improvements, Kenya is still not making sufficient progress in meeting the Millenium Development Goals targets [8]. The main functions of the health system against which performance is measured are stewardship, creation of resources financing and delivery of services [9]. Poor leadership has been identified as one of the key impediment to effective health system performance in Kenya. Additionally, devolution reforms have been identified to be highly complex and challenging to implement in similar settings [10, 11]. As a result, Kenya’s devolved system of government has exhibited leadership and governance challenges with regard to health service provision due to a radical departure from the highly centralized form of the governance system, leading to weak, unresponsive, inefficient, and inequitable distribution of health services in the country [12, 13]. These challenges, however, can be resolved through appropriate leadership training of healthcare workers [14]. Examples, where such ‘Leadership Development Program’ (LDP) training has been shown to result in improved health system performance, are provided in the cases reviewed. For example, the most recent Peterson and colleague cases relevant to our study [15, 16], is a study by Seims et al. [17] undertaken in Kenya prior to the devolution of the government functions. The findings revealed the positive impact of leadership training on the health service delivery indicators for the trained health workforce. The positive changes were sustained for 60 months after the leadership training. In contrast, there were minimal improvements in service delivery indicators in the comparison institution over the same period of time [17].

Another key study of interest was conducted in Upper Egypt by Mansour et al. [18]. Ten teams of health workers from five primary health units, three districts, one rural hospital and one team of governorate managers participated in the study. The team leadership challenge was to improve health services in three districts by increasing managers’ ability to create high performing teams and lead them to achieve results. The study results indicated the affirmative effect of the training on the following health indicators: a) reduction in the maternal mortality rate from 85.0 per 100,000 live births to 35.5 per 100,000 in Aswan Governorate; b) inspired and committed team changed from complaining about problems to identifying actionable challenges they could address and; c) when the results were tracked for 5 years, it demonstrated sustainability and scaling up [18].

Similarly, Kwamie and colleagues carried out a case study in Dangme West district in Ghana on the effectiveness of introducing LDP to Ghana’s district health system. The study further explored whether the program fostered systems thinking in decision-making by the district team. The study evaluated five teams from the district and sub-district hospital managers. Using a realist evaluation and theory of change, the team worked backward to determine the causal interaction between contexts, outcomes, and mechanisms. The findings suggested that the LDP training fostered the team’s initiative-building and improved prioritization resulting in the positive achievement of the short-term outcomes. Unlike Mansour et al. [18] positive sustainability results, the study reports lack of improvement in systems thinking due to the non-institutionalization of the LDP practices. The researchers concluded that when LDP was introduced in a complex system with semiautonomous features, chances are it tends to be rejected [19]. Utilizing the same approach but in a different context, Seddiq and colleagues analyzed 15 key informants’ care for TB patients’ centers in Afghanistan. The study was on the role of the leadership development program in restructuring the National Tuberculosis Control Programme (NTP) through the integration of tuberculosis treatment into primary health care and achieves most of its targets in conflict areas using the stop TB strategy by the year 2011. Using a case study methodology, the study findings revealed that the training was effective and performance measurements included: a) reduction in TB incidence from 325 to 189 and decreased mortality from 92 to 39 per 100,000; b) the program efficiency was credited to good governance by the government; c) the team reported strong leadership resulting to sound partnership and effective program stakeholder coordination; d) sufficient provision of funds and technical support from the development partners [20]. The above-highlighted evidence illustrates that a lot can be enhanced merely by effectively leading, managing and governing the existing health system resources especially the human resources for health [17–20].

Building strong and sustainable health systems requires a well-performing health workforce with the right competencies, attitude and capacity to offer quality health services using the limited available resources; this includes novel training for health workers [21]. In recognition of the wide range of health system strengthening strategies including leadership development, understanding how to design and implement effective leadership development for health is fundamental [22]. Although the potential impacts of leadership training among the health workers seem apparent, there are limited systematic inquiries of leadership development interventions and the practice of leadership development [23, 24]. Early literature evidence on coaching suggests positive effects of coaching in leadership development [24, 25]. Coaching, in essence, is defined as a process of supporting coachees to step back, and take in the “big picture,” and craft a future they desire through a commitment to the goal [26]. Team coaching consequently is a holistic approach for creating meaningful and lasting change for individual team members, the team as a whole, and the organization that the team serves [25]. Coaching is a means of enabling individuals or teams to clarify, prioritize and act towards improving performance through reflection and dialogue [27–30]. The role of the coach is to provide a unified team’s agenda and moderates conversations that foster teamwork towards a shared goal [28, 31, 32]. However, despite coaching popularity, research on coaching effectiveness is still limited [33, 34]. Few studies have evaluated the impact of health leadership training that includes coaching. Even with a rising number of well-designed studies in the area of leadership coaching, more methodical evaluations with appropriate criteria that connects theory and practice are considered necessary in leadership development tools [35, 36]. The degree to which leadership and management practices are internalized by an individual depends on the frequency and daily utilization of the newly learned skills and knowledge which are reinforced through coaching and mentoring [37].

For example, Grant used a (pre − post) design study to explore the impact of executive coaching during organizational turbulence such as change. His findings showed that coaching was associated with improved ability to deal with change and that the positive impact was generalized to non-work areas such as family life [28]. However, there was some criticism on the dyadic (one-to-one) coaching done in many organizations suggesting that interventions in organizations should also be targeted at the group level, as evidenced by the few existing models of group coaching that have been developed [31]. A comprehensive meta-analysis by Peters & Carr [32] on team effectiveness indicated that team coaching resulted in interpersonal, communication and improved team performance. The authors recommended that future researchers should conduct more management and leadership team coaching studies in real work settings. These are the research gaps that we sought to address.

### Leadership development training intervention

The current study is drawn from an ongoing leadership development program “Leading High-performing Healthcare Organizations” (LeHHO) designed for the health managers in Kenya. The program was co-created in 2010 by Strathmore Business School (SBS), Management Sciences for Health (MSH) and Ministry of Health, under the funding support by the United States Agency for International Development (USAID). The aim of the program is to foster the health manager’s application of leadership and management practices in order to resolve the persistent health service delivery challenges in Kenya. The program has been implemented in 6 cycles between the years (2010–2016) trained 165 leaders in Kenya. A critical part of the training is the incorporation of institutional improvement projects which have to be undertaken by teams from each institution sending participants for training. Between 2010 and 2016, 69 such projects have been undertaken by LeHHO trainees drawn from 39 health facilities in 19 counties in Kenya. The rationale behind the selection of the study program includes: a) The program was co-created by the key stakeholders in health as a post-devolution health system strengthening strategy; b) The LDP approach which integrates the classroom knowledge and application at the workplace through coaching is the unique leadership development approach with promising success; c) addressing universal health coverage challenges requires a multi-sectorial approach including capacity building across all the sectors (public, private and faith-based health facilities); d) programs which are deliberate planned with considerations on how monitoring and evaluation process can be easily evaluated; and; e) to the best of our knowledge, there is no impact evaluation of project-based experiential learning training that has been undertaken in Kenya post-devolution.

### Conceptual framework

**Fig. 1:**
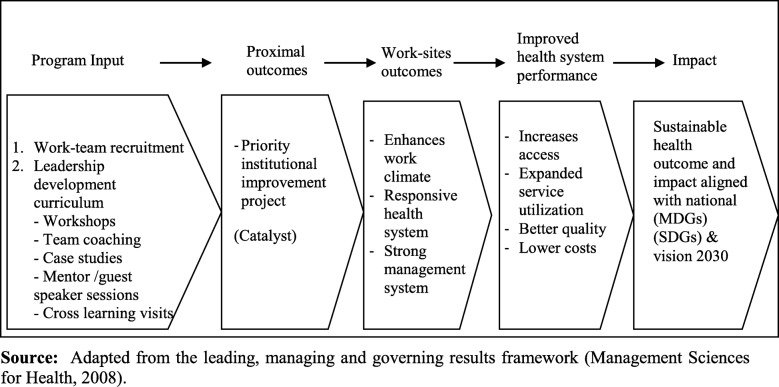
Depicted the project-based experiential learning model that combines the leadership, management and governance concepts from the Management Sciences for Health results model. Adapted from the leading, managing and governing results framework [39]

This study adopted the Management Sciences for Health “integrated leadership management and governance results framework”. The framework integrates leading, managing and governance practices approach towards developing managers who lead and govern well. The program is anchored on the assumption that “leadership can be learned through an action learning approach where participants learn to apply a set of leading, managing, and practices to address their real workplace challenges over time” [38]. The program’s design unique feature includes its ability to challenge the participants to learn and apply leadership and management practices through the application on real health service delivery challenges. The LMG approach operates on a framework that links the newly acquired knowledge and skills to current challenges facing health managers in their workplaces through the implementation of action plans. The project-based experiential learning model (Fig. 1) utilizes the MSH results model which illustrates the program principle that measurement of leadership, management and governance capacity is an integrated process [40]. The model suggests that the application of the leadership, management and governance practices potential results in improved work climate, sound management, and transparent governance system, and ultimately improving health service delivery [40]. The results model was espoused since it combines the interconnected learning activities and the intended outcome of the LeHHO program presented in this paper. It was anticipated the trained health managers would demonstrate improvement in health service delivery indicators.

#### Program delivery approach

The aim of the LeHHO program is to enable senior health managers drawn from different levels and sectors of health service to plan and implement their organization strategic plans, through prioritization of one challenge at a time. The program cohort cycle is implemented within a nine-month period and composed of; five workshop modules; four team coaching sessions and one cross-learning site visit. Each workshop module is equivalent to four classroom days, and a coaching session takes between 60 to 120 min. Unlike mentorship which is holistic involving hand-holding and provision of answers based on experience, coaching is largely utilized in LeHHO program because it involves an active process of imparting specific skills to the coachees that enable them to achieve a particular goal. The coaching session acts as a link between (a) the classroom learning; (b) the application of the learned knowledge in the workplace; and (c) team support and accountability. Trained local and international faculty and coaches were seconded by the local facilitators to deliver the training. In line with the needs of the participants as experienced managers, the primary focus of delivery was “participant-centered learning” (Koyoson, H: Senior Healthcare Management Program Curriculum: Strathmore University Business School, Institute of Healthcare Management, unpublished) . This type of learning is particularly suitable to the target audience in that it has as its core ingredient that combined experiences of the team participant. The teaching methodology included: case method, experiential learning, and group work. At the end of the program, the participants were expected to present their project implementation progress to their peers and the program facilitators for feedback.

#### Integration of team coaching into the leadership development program

The LeHHO program brings together key healthcare industry stakeholders in Kenya to promote unity focus on the ‘patient’ irrespective of the health sector. Team coaching interventions allow integration of learned skills directed to health system performance indicators. The approach focused on teams and systemic culture transformation within the healthcare setting. The principal objective of team coaching is to link the program’s modules back to workplace goals while fostering tangible, sustainable results and relationships. The expected output from team coaching dialogue is an attitudinal shift for behavioral change, and the development of a universal framework about the key elements of high performance such as shared vision, constant application of best practices and norm [41]. The overarching objective of team coaching is to link experienced health professionals across the health sectors (public, private & faith-based), to share knowledge and experiences hence building their leadership capacities, and develop a pool of coaches and mentors within the public sector for sustainability. The team coaching module is aimed at developing and sustaining quality health workforce performance. This is an effort towards supporting Kenya’s healthcare strategic goals and ensures mission success in the future of developing a versatile and competent workforce to meet the long-term needs of healthcare institutions. Team coaching, therefore, serves as a knowledge and skill transfer tool developed to foster a positive work climate while encouraging a strategy that guides the workforce to produce tangible results. The target teams for coaching modules are all LeHHO participants interested in growing more leaders across the organization while addressing one challenge at a time and achieving desired measurable results. One coaching session is approximately 2 h. The project teams scanned their work environment and identified a key challenge area to focus on; the teams were then randomly matched with coaches by the end of the module during coach and coachee formal introduction session. The challenge model is introduced to the participants during module 1 of the workshop and forms the ‘heart’ of the leadership learning and application plan as well as coaching conversation guide throughout the training. The Challenge Model [40] is a logical approach that enables teams to plan and solve worksite problems through the implementation of the action plan. The purpose of using the Challenge model was to identify priority institutional improvement projects. These projects were aligned to Institutional Strategic Plans. It is these projects that provided a platform for coaching. The coaching sessions are interspersed with the workshop modules. The challenge models were filled by the project teams at the beginning of the program and the action plan was developed around the challenge model’s priority actions. For learning and accountability purposes, the project teams presented and received feedback on the progress of their projects from the class at the beginning of every program module. The project indicators at the beginning of the training were labeled as a baseline while the project indicators at the end of the training were labeled as endline. The aim of this study was to assess the impact of project-based experiential learning on health service delivery indicators. Unique from the related existing studies by; Seims et al. (2012), Mansour et al. (2010), Kwamie et al. (2014) and Seddiq et al. (2014), [17–20]. The focus of the study was on the role of senior health managers who are the policymakers in the public, faith-based and private health institutions. Indeed, the element of coaching support was highlighted in these studies, however understanding the perceived effect of coaching on the results of the project is warranted. The study findings contribute to the empirical literature on how incorporating institutional improvement projects and coaching into leadership training leads to the immediate application of knowledge into health system performance improvement in Kenyan Counties.

The current study is part of larger research investigating the “impact of leadership development on sustainable health systems performance”. Unlike our current study which focuses on the service delivery pillar, the broad study focused on the impact on the six pillars of the health systems performance (service delivery, leadership, and governance, information, financing, human resource for health, medicines, and technology). For this study, we exclusively focused on the service delivery pillar. The main objective of the present study was to investigate the effects of integrating challenge projects and team coaching into leadership development training on health service delivery indicators in selected facilities in Kenya. The health outcome indicators of interest included; skilled birth attendance, full immunization, service quality improvement and increased utilization of health services at the facility. The current study makes three significant contributions to this objective. First, we assessed the impact of training intervention on the selected service delivery indicators by comparing the indicators of interest for the intervention and a control group of the same time period (pre-test and end of training). Second, we evaluated the sustainability for both the intervention and control group by comparing the service delivery indicators at the post-test (end of training and 24–60 months post-training). Third, we examined the team’s perceived contribution of team-coaching towards the improvement of the service delivery indicators. The projects were used as a unit of analysis for measuring the effectiveness of leadership training.

## Methods

### Study design and setting

The study utilized a quasi-experimental design. The effectiveness of the training program was assessed using a pre-test and post-test design. The design was suitable for the study because it served to reinforce causal deduction and decreased ethical dilemmas. Additionally, the design is considered more effective for un-controllable environmental events [42]. The study was conducted in health facilities from the following Counties; Nairobi, Kajiado, Kiambu, Mandera, Elgeyo Marakwet, Kisumu, Samburu, Nakuru, Busia, Kisii, Siaya, Uasin-Gishu, Kakamega. Health facility managers were recruited through an expression of interest process addressed to the LeHHO alumni for the treatment group and the Medical Superintendent for the control group. The study targeted health managers who had undergone the LeHHO training from the public, private, faith-based and non-governmental health institutions. Between the years (2010–2016), LeHHO program had trained a total of 165 county health leaders from 19 counties. The leaders were trained to acquire and practice leadership knowledge, skills and practices in the work environment during and post-training. During the training, the 165 participants formed teams that undertook 69 institutional improvement projects aligned to county or institutional strategic plans. These projects were housed in 39 health facilities within 19 Counties in Kenya and, 15 of the projects from 13 counties focused on service delivery improvement pillar. The intervention project teams were purposively sampled. These teams were assigned as the treatment group. The sample selection was informed by the project category in reference to the six health system pillars. The control group comprised 14 other health institutions within the same counties that were selected and matched with the intervention group within the same county. The rationale for their matching was that both facilities were operating within the same county health system and guided by a common strategic plan. The purpose of the study, as well as voluntary participation, was explained to the key informants. Written informed consent was obtained from participants before the interview session. Pre-test and post-test data were collected in three-point periods (beginning, end of the training, and 24-to-60 months post-training).

#### Service delivery indicators in intervention (leadership training) and comparison health facilities

In order to ascertain changes attributed to leadership training, data was collected from 15 intervention and matched with 14 comparison institutions within the same county. Consideration of the selection of the intervention and comparison health facilities was based on the service delivery coverage and informed by the same county strategic plan. Fifteen intervention facilities were purposively selected and matched on with the comparison facilities within the same county as summarized in (Table 1).

**Table 1:** Presents a summary of the number and percentage of the service delivery indicators of interest in the intervention and control health facilities per health sector

Indicator	Health sector	N & % (intervention hospitals)	N & % (comparison Hospitals)	Total N & %
Skilled deliveries by birth attendants.	Public	4 (26.6%)	4 (28.7%)	8 (27.6%)
Full immunization of children	Public	1 (6.6%)	1 (7.1%)	2 (6.9%)
Increased outpatient & inpatient utilization	Private, public & faith-based	4 (26.6%)	3 (21.4%)	7 (24.1%)
Reduce outpatient turn-around time (TAT)	Private, public & faith-based	2 (13.3%)	2 (14.3%)	4 (13.8%)
Others (increase quality and customer satisfaction	Private, public & faith-based	4 (26.6%)	4 (28.6%)	8 (27.6%)
Total		15 (100)	14 (100)	29 (100%)

Source: Survey data 2018

#### Data collection and analysis

The study utilized both primary and secondary data. Primary data was collected using questionnaires. The questionnaires comprised of close-ended questions aimed at providing structured responses to the study’s outcomes (Additional file 1). The questionnaire contained questions on the stated institutional improvement targets for the projects and covered the baseline, endline and post-training measurements for the selected health service delivery indicators. The questionnaire took an average of 12 min to complete. To enhance the validity and accuracy of data, questionnaires were piloted on four team-based projects within two counties before being used. A total of 15 project team leaders were sent a soft copy of the close-ended questionnaire and they were requested to fill the questionnaires in September 2018. The initial survey response rate was 64%. A follow-up survey prompt was sent to non-respondents, hard copy questionnaires were sent through mailing postage response envelope resulting in a response rate was 100%. Secondary data were drawn from the team’s challenge model documents which were filled in at the beginning of the training and the end of the training. Additional secondary data in the form of routine program module and cohort reports for the 6 years of the project duration were used for references. These documents were retrieved from Strathmore Business School Institute of Healthcare Management database. The control team indicators data were collected in October 2018 with the assistance of health management information system officers from Kenya’s ministry of health (MOH). The data was only on the health service delivery indicators corresponding with the baseline, endline, and post-training for the intervention group project period. In the case of incomplete or missing data, the managers from the control facilities were contacted for support. Data were entered, cleaned and analyzed in Microsoft Excel and significance tests calculated using the statistical package SPSS version 20. Descriptive statistics and paired-sample t-test analysis were used to show the relationship between dependent variables (improved service delivery indicators) and the independent variable (the training intervention). A significance level of .05 was set for the tests.

## Results

The study findings are presented in 3 sub-sections: a) LeHHO team leader’s demographics; b) impact and sustainability of training intervention on the health service delivery indicators at the end of training period (9 months) for the intervention group compared with control group and; c) the participants perceived effect of team coaching on the priority projects.

### Socio-demographic profiles

Fifteen health managers participated in the study. There were 9 (60%) male and 5 (40%) female participants in the study. Seven (47%) of the participants were between the age category of 46–55 years. Eight (53%) of the participants had a master’s degree educational level, implying there is a reasonably high level of tertiary education (Table 2). This is a reflection of Kenya’s healthcare manager’s recruitment and promotion practices are based on the attainment of higher educational qualifications.

**Table 2:** Illustrated the study participant’s socio-demographic information (gender, age, and education)

Item	Category	Frequency & Percentage (%)
Sex	Male	9 (60%)
	Female	6 (40%)
Age Category	26-35 yrs.	1 (7%)
	36- 45 yrs.	5 (33%)
	46-55 yrs	7 (47%)
	> 55 yrs.	2 (13%)
Highest Education Level	Bachelor degree	6 (40%)
	Master degree	8 (53%)
	Others	1 (7%)

Source**:** Survey data 2018

### Impact and sustainability of training intervention on the project’s indicators

**Fig. 2:**
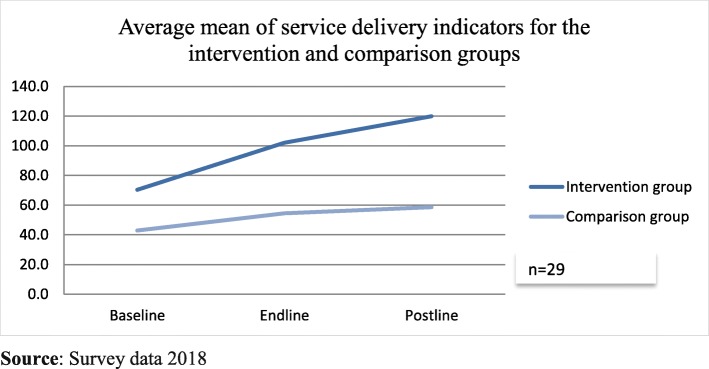
Illustrated the trend comparing the means from baseline, endline and post-training measures for the 15 intervention team projects and the control team respectively

A total of 15 service delivery improvement projects were prioritized by the teams as aligned to their strategic plan. Out of the 15 projects [14], 93% of the prioritized projects achieved the desired measurable results (DMR) by the end of the training (9th month). Eighty percent of the implemented projects were sustained over time, the post-training data represented the state of indicators of interest for data collection at the time of the study data collection (August 2018). The trend of means from baseline, endline and post-training measures for the 15 intervention group project were (70.4, 102.1 & 119.8), while the control teams means were (42.9, 54.6 & 58.7) respectively. The comparisons of the means are summarized in (Fig. 2). Data were further analyzed using paired-sample t-tests and the results are summarized in (Table 3). The *p* values for the intervention group comparing the; baseline to endline was (0.017), and (0.061) for the endline to post-training. For the comparison group, the p values for the baseline to the endline was (0.173), and (0.095) for the endline to post-training. These results imply that the trained teams’ indicators improved significantly as compared to the non-trained teams. These findings suggest that participation in the LeHHO program was associated with a significant increase in priority project goal attainment.

**Table 3:** Presented the paired-sample t-test comparing baseline, endline, and post-training for the trained and non-trained teams

	Paired Differences							
	Mean	Std. Deviation	Std. Error Mean	95% Confidence Interval oDifference		t	df	Sig. (2-tailed)
				Lower	Upper			
Comparison group								
Baseline-endline	−11.7000	31.5220	8.1389	−29.1563	5.7563	−1.438	14	.173
Endline-post-training	−4.1000	8.8645	2.2888	−9.0090	.8090	−1.791	14	.095
Intervention group								
Baseline-endline	−31.6000	45.0267	11.6258	−56.5349	−6.6651	−2.718	14	.017
Endline-post-training	−17.8333	33.9557	8.7673	−36.6374	.9707	−2.034	14	.061

Source: Survey data 2018

### Perceived effect of coaching on project implementation

The findings on the perceived effect of coaching on the implementation of priority projects revealed that the participants highly attribute the success of their project to coaching. Ninety-three percent of the study participants reported that the achieved project results were highly attributed to the challenge model team coaching approach.

## Discussion

The current study extends the healthcare workforce leadership development literature, that demonstrates the efficacy of conducting a leadership development intervention that integrates catalyst projects with coaching. The study found that the LeHHO program contributed to the improvement of the health service delivery indicators in the selected health facilities as demonstrated by the mean indicator score change at pre-test and post-test. Overall, the findings support the LeHHO theoretical framework which was adapted from the Management Sciences for Health (MSH) the Leadership, management and governance (LMG) model. The assumptions of the LeHHO training approach suggest that exposing health managers to a training activity (workshop, case studies, team recruitment, challenge model approach, and team coaching), influences participants’ ability to apply the learned knowledge and skills at the real work environment. The findings further imply that the selection of catalyst projects according to priority areas for improvement within the health facility by health managers triggers the immediate application of knowledge to the work environment. It is anticipated that the implementation of the catalyst projects will ultimately result in improved health systems performance. The projects, therefore, provided the basis for direct measurement of leadership development effect because its contribution to tangible results can be measured and monitored. This is evident in the high project completion rate of which of the prioritized projects achieved the desired measurable results (DMR) by the end of the training (9th month). The trend of means from baseline, endline and post-training measures for the 15 projects as compared to the control group means confirms progressive improvement in the indicators. This is because the trained teams achieved higher means than the non-trained teams. These findings echo Salas et al. [43] work which outlined that the evidence in changes in health systems strengthening through leadership training and team training approach is associated with positive changes in health service delivery.

In reference to the most recent comparable leadership development study on the impact of leadership on organizational performance in Kenya, Seims and colleagues’ findings revealed the positive impact of the leadership development training on the selected health service delivery indicators. Additionally, their study further demonstrated that there was positive sustainability and scaling up of the positive results beyond the training. In addition to their findings, an important new contribution that the current study makes beyond the previous similar studies lies in the context of Kenya’s devolved health services and the role of health manager during the change process such as health reforms. Again, Seims et al. [17] study examined frontline health workers in public health facilities before the devolution of the health services in Kenya. More precisely, the present intervention has shown to influence the health services indicator not just for the public health facilities, but also in the private and faith-based health facilities. This is a significant contribution to the literature because the study examined the effects of the leadership development program in a devolved system across the health sectors. This suggests that implementation of an integrated leadership intervention does not only affect the dynamic public health facilities which were directly affected by the devolution of health services but can also affect health facilities where there are different governance structures and are indirectly affected by the devolution process. These findings have a proposition for Kenya’s health system performance improvement in the short and long term.

The current study specifically focused on healthcare management teams who exclusively prioritized and implemented a health service delivery catalyst project. The teams were from different cohorts for the years 2011–2016. The impact and sustainability rate for the implemented projects propose that participation in the LeHHO program was associated with significant increases in priority project goal attainment for the intervention group as compared to the control group. These findings suggest that exposure to the LeHHO training resulted not only in improved indicators but also result in the sustainable outcome to up to 60 months after the training. Additionally, there was some indication that the training also fostered teamwork and a positive work climate during project selection and implementation. Even though the impact of training on teams and work climate was not entirely assessed in this study, it is a potential area for future research. These results support findings from studies by [15, 17–20]. This shows that the LeHHO program approach could be a useful experiential training design for implementing sustainable leadership capacity building interventions for the health workers in a low-income setting. The study findings are extremely important by contributing to the incremental knowledge particularly in a poorly governed health systems resulting in unsustainable health outcomes, despite the immense investment in health system strengthening interventions in Africa.

One perceived key success factor for the LeHHO program training approach was team-based coaching crafted around the institutional improvement project. The 9-months training included a series of four coaching sessions interspersed in between the modules to facilitate the team’s project implementation at the workplace. The majority of the participants attributed the success of their projects to team coaching sessions. Prioritization and implementation of institutional improvement projects using the challenge model was a unique characteristic that distinguished the LeHHO program. The projects encouraged the healthcare managers to identify a challenge related to their workplace through the application of the learned leadership skills on real workplace challenges. The coaching sessions were meant to challenge the coaches to scan and focus on a catalyst priority project which was within their influence and control. Indeed, Carey et al. (2011) [44] and Peters et al. (2013) highlights the importance of team coaching in providing an objective view of the team and facilitates conversations that enable the team to adjust their ways of working together in service of their goals [25, 32]. Team meetings facilitated shared vision among team members, foster teamwork and commitment towards addressing one challenge at a time. A similar approach confirms leverage on team members’ unique talents and foster team building, which is important in organizational performance [43]. The cases reviewed by Peterson et al. (2011) & Hatt et al. (2015) present examples of where leadership development program training which incorporates coaching around priority projects, has resulted in improvement of health system performance [15, 16]. However, these earlier studies focused on evaluating the impact of the leadership training on the health service delivery indicators and sustainability but did not explore the role of coaching even though it was part of the training curriculum in the similar programs. This study addresses this gap. We explored participant’s perceived contribution of team coaching as part of the training curriculum. The current study places coaching around the catalyst project at the center of the LeHHO training curriculum because the project is the study unit of analysis. Consistent with the growing body of literature on coaching as a leadership development tool, Carey et al. (2011) [44] & Peters et al. (2013) presents the benefit of team-based coaching in leadership training [25, 32]. The study findings suggest that the LeHHO program could have the feasible potential of improving health outcomes in low-income countries.

### Limitations

The following limitations were identified in this study. First, the setting and point of data sources were exclusively from the Strathmore healthcare leadership program. This limits the findings to the program alumni and should be generalized with caution. Second, due to the longitudinal nature of the study, the quality of information may not represent the current state of institutional leadership due to frequent change in leadership positions. However, follow-up calls and emails were sent to alumni who were transferred or retired to sense check the team results. Third, the intervention and comparison group facilities were not randomly selected, which could lead to possible bias. Nonetheless, the comparison facilities were informed by the same county strategic plan. Lastly, because coaching as a leadership development tool is a fairly new phenomenon in the healthcare context, there was limited background literature on the integration of coaching into leadership training. However, case studies from the Management Sciences for Health ‘Leadership Development Program’ (LDP) offer a solid ground to draw project experiences.

## Conclusion

The study findings contribute to a series of integrated evidence-based approaches to enhance the transfer of leadership, management, and governance practices through team coaching conversations for inspired commitment to problem-solving. The findings show that the achievements of priority project goals are positively influenced by the integration of both teaching and application of practices. The results also suggest that incorporating institutional improvement projects and coaching into leadership training leads to the immediate application of knowledge into Health System performance improvement. Therefore, low and middle-income countries like Kenya need to invest in leadership and coaching training for health workers, together with the strengthening of other health system pillars (information, financing, human resource for health, medicines and technology, and service delivery) for sustainable health systems performance improvement to be realized. These findings suggest that LeHHO alumni should integrate coaching in each work environment through a partnership of the training institutions and health management teams. This is also aligned with recent systematic reviews on factors that influence positive results of such training as self-management and relapse prevention strategies. It is therefore recommended that organizations should design programs that integrated knowledge transfer and maintenance during and post-training. This approach will facilitate sustainability and scaling up of the achieved results, and institutionalization of coaching practice across the organizations.

## Supplementary information

**Additional file 1.** Quantitative Research Instrument.

## Data Availability

The data utilized for this study can be made available from the first author upon reasonable request.

## Abbreviations

DMR: Desired Measurable Result
HSS: Health System Strengthening
LDP: Leadership Development Program
LEHHO: Leading High-Performing Organisations
LMG: Leadership, Management, and Governance
MSH: Management Sciences for Health
SBS: Strathmore Business School
SPSS: Statistical Package for Social Scientists
USAID: US Agency for International Development
