# Supplementary material for: Effect of project-based experiential learning on the health service delivery indicators: a quasi-experiment study
Source: BMC Health Serv Res. 2020 Feb 26;20:144. doi: 10.1186/s12913-020-4949-5 (PMC7045382; doi:10.1186/s12913-020-4949-5)
Supplement: Supplementary file 1 — Additional file 1. Quantitative Research Instrument. [file 12913_2020_4949_MOESM1_ESM.docx]

Quantitative Research Instrument

**Section 1: Demographics**

| Description | During Training | Current |
| --- | --- | --- |
| Name of organisation |  |  |
| Name of respondent |  |  |
| Position of respondent |  |  |
| Years worked in the organisation |  |  |
| Years worked in current position |  |  |
| Type of organisation |  |  |
| County |  |  |

1. Sex:

- - Male
  - Female

2. Which age category do you belong to?

- 18-25 yrs
- 26-35yrs
- 36- 45yrs
- 46-55yrs
- Over 55yrs

3. What is your highest educational qualification?

- Bachelors degree
- Masters degree
- Doctoral degree
- Others … (Specify)

4. How many other leadership training did you attend prior LeHHO Program? No........

Please list them down

1. ......................................................................................................
2. ......................................................................................................
3. ......................................................................................................
4. ......................................................................................................

**Section 2: Leadership Priority project desire measurable result status**

| **Institutional Improvement Priority**  **Challenge** |  | |
| --- | --- | --- |
| **Measurement Indicator** |  | |
| **Desired Measurable Result (DMR)** |  | |
| **Baseline Indicator measure** | **Endline indicator measure** | **Post-training indicator**  **measure** |
|  |  |  |
|  |  |  |
|  |  |  |

**Section 3: Role of Coaching towards Achievement of Organisational Improvement Priority Challenge Project.**

| **Checklist**  Please complete the following coaching evaluation by rating each item on a scale from 1 to 3 **where:**  1= Below expectations 2= Above expectations 3= Don’t know | | | | |
| --- | --- | --- | --- | --- |
| 1 | Overall how would you rate the contribution of leadership training alone on the achievement of project results? | 1 | 2 | 3 |
| 2 | How well did the leadership coach relate to your institutional environment? | 1 | 2 | 3 |
| 3 | How effective has the coaching process impacted on actual organizational results? | 1 | 2 | 3 |
| 4 | How effective was your leadership coach in assisting you and the team to identify your institutional priority project goals and objectives? | 1 | 2 | 3 |
| 5 | How effective was your leadership coach at supporting you to achieve your identified priority project goals and objectives? | 1 | 2 | 3 |
| 6 | How effective was the challenge model as a guiding tool in the coaching conversations towards achieving desired results? | 1 | 2 | 3 |
| 7 | How effective were coaching outcome to your personal life? | 1 | 2 | 3 |
| 8 | How effective were the coaching outcome impacting your organization? | 1 | 2 | 3 |
| 9 | How would you rate the number of coaching sessions you had? | 1 | 2 | 3 |
| 10 | How would you rate the long session of the coaching sessions? | 1 | 2 | 3 |
